# Rapid Screening of Complex DNA Samples by Single-Molecule Amplification and Sequencing

**DOI:** 10.1371/journal.pone.0019723

**Published:** 2011-05-19

**Authors:** Jiaqi Huang, Zongli Zheng, Anders F. Andersson, Lars Engstrand, Weimin Ye

**Affiliations:** 1 Department of Medical Epidemiology and Biostatistics, Karolinska Institutet, Stockholm, Sweden; 2 Department of Microbiology, Tumor and Cell Biology, Karolinska Institutet, Stockholm, Sweden; 3 Science for Life Laboratory, School of Biotechnology, KTH Royal Institute of Technology, Stockholm, Sweden; 4 Swedish Institute for Communicable Infectious Disease Control, Solna, Sweden; Argonne National Laboratory, United States of America

## Abstract

Microbial cloning makes Sanger sequencing of complex DNA samples possible but is labor intensive. We present a simple, rapid and robust method that enables laboratories without special equipment to perform single-molecule amplicon sequencing, although in a low-throughput manner, from sub-picogram quantities of DNA. The method can also be used for quick quality control of next-generation sequencing libraries, as was demonstrated for a metagenomic sample.

## Introduction

**Figure 1:**
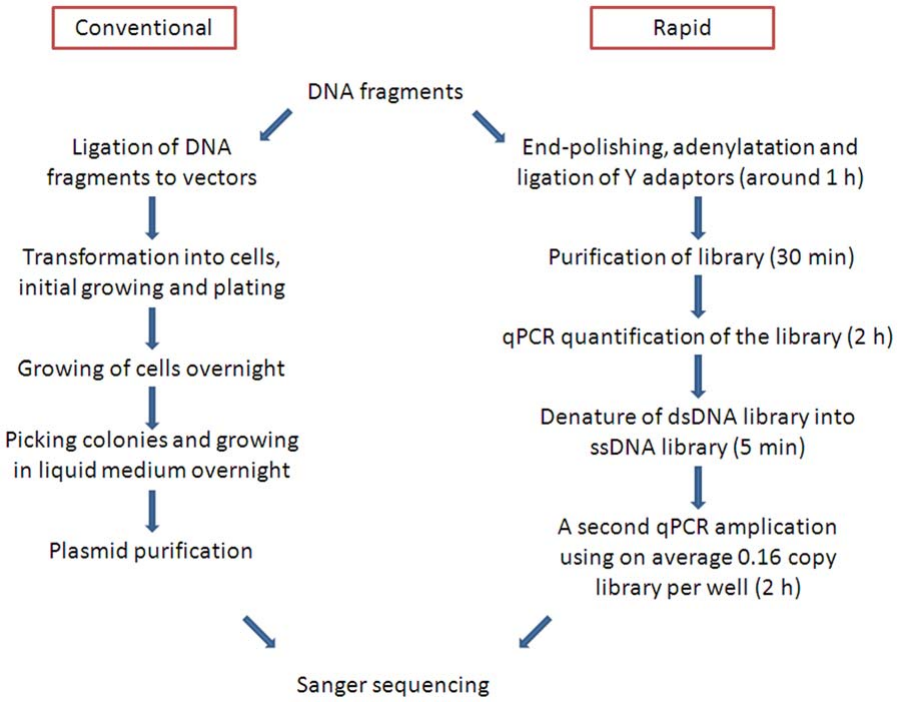
Conventional way of sample preparation for Sanger sequencing, in comparison with our rapid method.

Sanger sequencing has long been, and still is, the most used method for DNA sequencing. It has, no doubt, been seminal in the development of modern genomics [1]. Applying Sanger sequencing on complex sample materials commonly requires an initial microbial sub-cloning step, which is also often used for validating next-generation sequencing libraries [2]. Microbial sub-cloning is a tedious process that involves bacterial culture, selection of positive colonies and purification of plasmids (Figure 1), and it often requires substantial amounts of starting material. Alternatively, a dilution method followed by PCR to generate polonies (polymerase-colonies) has been proposed for somatic mutation analysis of single cells [3]. However, this targeted approach using primers for amplifications of specific loci cannot be directly adjusted to screen a complex DNA population of unknown sequences. Further, handling of complex DNA at the single DNA molecules level, rather than at the single cells level, remains challenging due to the infinitesimal mass involved. When the sample mass is extremely small, several factors, which can usually be ignored in sequencing at the single cell level, start to come into play.

Recent developments in micro-fabricated devices and microfluidic systems have facilitated high-throughput amplification of DNA molecules in parallel. Microfluidic Sanger sequencing has been used to dramatically save reagent cost and to take advantage of the long reads Sanger provides [4], but has not yet reached the single-molecule level. New technologies for high throughput single-molecule amplicon Sanger sequencing are under development [5], but are not available for most laboratories.

Here we present a readily available, simple and robust method for performing rapid single-molecule amplification and Sanger sequencing of trace amounts of starting materials. The method mainly involves ligation of a universal adapter that is resistant to exonuclease digestion, use of a zero absorbance tube, and quick identification of positive polonies by a Taqman assay. The method is an appealing alternative, feasible for laboratories without special equipment, to the commonly used microbial sub-cloning assay, with the additional advantage of being applicable on sub-picogram levels of DNA. It may also be useful when the DNA constructs are toxic to the microbial host.

## Materials and Methods

### Library construction

DNA libraries were prepared using a Y adapter, as described previously [6]. In short, genomic DNA was nebulized, end polished, adenylated and ligated with the Y adapter. Purified library was eluted in 25 µl of 1 X TE buffer and quantified using a Taqman MBG probe standard curve assay. Libraries were stored in low binding tube (pollyallomer, Beckman Coulter). Immediately before use, libraries were denatured into single-stranded templates. The oligonucleotides sequences are: Y adapter top 5–C*C*A*T*C*T*CATCCCTGCGTGTCTCCGA*C*T*C*A*G*T-3; Y adapter bottom 5-pC*T*G*A*G*TCGGACACGCAACAGGGGATAGaCAAGGCACACAGGG*G*A*T*A*G*G-3 (* denotes a phosphorothioate-modified bond; p denotes a phosphorylation, IDT technology, HPLC purification). The Y adapter was formed by incubation at 95°C for 1 min, 60°C to 15°C with −0.1°C per second, 14°C held.

To assess the background noise introduced by adapter dimers, various amounts of adapters (1 µl of 1 µM, 0.1 µM, 0.01 µM and 0.001 µM) were used for library construction without adding samples. Next, a mock size selection step was included to select for 300-bp to 900-bp long fragments in a similar way as for purification of true sample library [6], and eluted in 25 µl 1 X TE buffer. Two microliter was used for quantification using a qPCR standard curve assay [6].

To investigate the lowest possible amount of starting DNA, nebulized DNA (*H. pylori*) having a median size of ∼500 bp (range 300 to 900 bp) was measured using Qubit fluorescence system (Invitrogen) and diluted into 55 picogram, 550 femtogram, 550 attogram and 550 zeptogram per microliter, corresponding to 100 million, 1 million, 1000 and 1 copies of DNA molecules per microliter, respectively.

### Generation of polonies

According to the Poisson distribution, sample concentration should be kept low to achieve a high proportion of single-molecule polonies among all (Table S1 and Figure S1). We chose a concentration of 0.16 copy of amplifiable template per reaction on average with the expectation that 92% of polonies would be derived from single-molecules. qPCR was carried out in a 10 µl final volume for the 96-well plate, or 5 µl for the 384-well plate, containing 1 x Taqman Fast Universal Buffer (Applied Biosystems), 900 nM primer emPCR.Fwd, 900 nM primer emPCR.Rev, 200 nM Taqman probe and 0.16 copy of template per well. The cycling condition was incubated at 95°C for 3 min; 45 cycles of 95°C for 15 sec, 60°C for 1 min; 68°C for 1 min; 4°C held, on the 7900HT Real Time System (Applied Biosystems). The primer sequences were: emPCR.Fwd 5′-CCATCTCATCCCTGCGTGTC-3; emPCR.Rev 5′-CCTATCCCCTGTGTGCCTTG-3′; Taqman probe 6FAM-CTATCCCCTGTTGCGTGTC-MGBNFQ (Applied Biosystems). All the primer sequences used in this study had been published previously [6].

### Sanger sequencing

Sequencing reaction was performed in a 20 µl final volume containing 3 µl of BigDye Terminator v3.1 Sequencing Buffer (5X), 0.32 µl of 10 µM emPCR.Fwd primer, 2 µl of BigDye Mix and 1 µl of qPCR polony products (the wells with the green ‘Pass’ flag). The sequencing reactions were then purified using Sephadex according to the manufacturer's instruction and followed by Sanger sequencing.

## Results and Discussion

Our method for sub-cloning free Sanger sequencing (outlined in Figure 1) is based on ligation of universal Y-adapters to fragmented, polished and adenylated DNA fragments, quantification of total library by Taqman MGB, distribution of the library into 96- or 384-well plates at an average concentration of 0.16 PCR amplifiable library molecules per well, and Taqman MGB to identify positive polonies that will subsequently be sequenced.

In a ligation reaction to generate adapter-template products, side product adapter dimers may form and introduce noise. Although adapters are designed to have overhangs to prevent adapter-adapter formation, an ultra-accurate oligonucleotides synthesis cannot totally prevent the formation of adapter dimers. For instance, 1 µl of 1 µM adapters with 99.9999% synthesis accuracy (one error out of one million) means 6^5^ copies of erroneous adapters. Taking into account the length of the adapter (∼50 bp), these 6^5^ copies of erroneous adapters means many thousands of adapters that have erroneous ends and perhaps lack functional overhangs. Consequently, these erroneous adapters may form adapter dimers in the ligation reaction. Even though a size exclusion step is included to remove fragments shorter than 300 bp which should remove adapter dimers (<100 bp), a very small remaining subset of adapter dimers may be overwhelming in the context of single molecule template. The adapter dimer problem is well-known in the sequencing community (see e.g. seqanswers.com). We thus tested the level of background noise introduced by different amounts of adapter dimers in reactions without sample templates. Table 1 shows that, in our experimental setting, 2 µl of no template library (total 25 µl) prepared by 1 µl of 1, 0.1, 0.01 and 0.001 µM adapters had a Ct value of 29.5, 32.9, 37.8 and undetermined, respectively.

**Table 1:** Background noise tests of adapter dimers at different concentrations and their resulting Ct values.

Adapter concentration, µM (1 µl)	Ct values (mean)
1	29.5
0.1	32.9
0.01	37.8
0.001	Undetermined

Based on these results, we selected adapter concentrations of 0.01 and 0.001 µM to test the lowest possible amount of starting sample. With an adapter concentration of 0.01 µM, the assay could distinguish signal from 1 million copies of templates (Ct 35.7) from background (Ct ∼38, see the background test in Table 1 and 1 copy template in Table 2). With a lower adapter concentration at 0.001 µM, which gave no background signal, the assay could detect 100 million copies of templates.

**Table 2:** Lowest detectable amounts of starting sample templates using 1 µl of 0.01 and 0.001 µM adapters.

Adapter concentration, µM (1 µl)	Template, copies (∼ gram of a 500-bp dsDNA)	Ct value, mean
0.01	100 million, (∼55 pico-)	26.9
0.01	1 million, (∼550 femto-)	35.7
0.01	1000, (∼550 atto-)	37.8
0.01	1 (∼550 zepto-)	38.2
0.001	100 million, (∼55 pico-)	35.5
0.001	1 million, (∼550 femto-)	Undetermined
0.001	1000, (∼550 atto-)	Undetermined
0.001	1, (∼550 zepto-)	Undetermined

**Figure 2:**
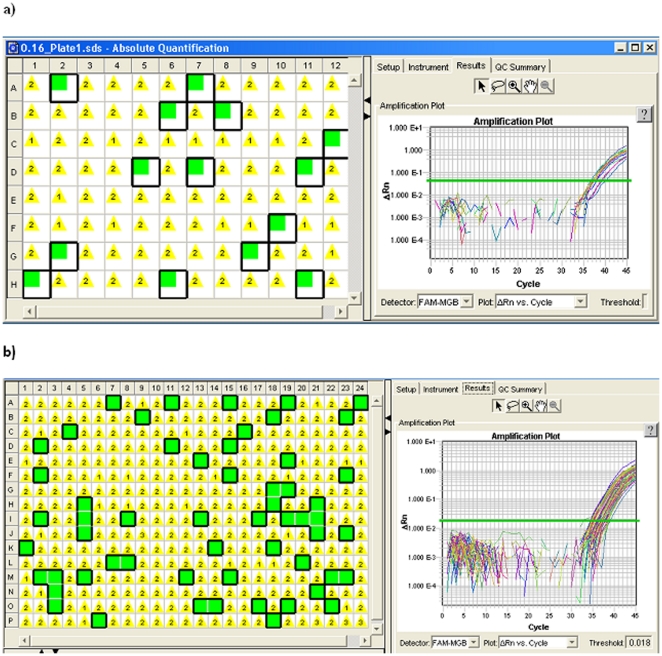
Generation of polonies and visualization by qPCR. The green wells (Pass flag) showed 14 and 57 polonies in a 96-well plate **(a)** and a 384-well plate **(b)**, respectively.

Before generating polonies, absolute quantification of the library was performed using a Taqman MGB-based qPCR standard curve assay [6], using an MGB probe targeting a sequence in the Y-adapter. To obtain a high proportion of polonies derived from single-molecule, sample libraries were diluted into 0.16 copy per well (Table S1 and Figure S1). The library prepared from 100 million copies of DNA fragments (*H. pylori*) and 0.01 µM adapter was quantified and diluted into 16 copies of PCR amplifiable library molecules per µl. One microliter was then added into a qPCR master mix for 100 reactions. Consistent with the Poisson distribution (which predicts 13.1 single-molecule polonies and 1.1 mixed-molecule polonies), 14 wells turned out positive (Figure 2), yielding 13 Sanger reads and 1 failed. These 13 reads were confirmed as *H. pylori* DNA by comparing (BLASTn alignment) with the NCBI database. These sequences had different gene features (no duplicated polonies). The median size was 378 bp (range 118 to 525 bp) and the median alignment identity was 98% (range 95% to 99%; see Data S1 for BLASTn alignment details).

To screen a metagenomic sample from sea water, we applied this method and generated 57 polonies (predicted by Poisson: 52.4 single-molecule and 4.4 mixed-molecule) using a 384-well plate, yielding 44 Sanger reads and 13 failed. Among the 44 reads, 37 were mappable to the NCBI database and one additional was identified by mapping to assembled contigs of this metagenomic library (which was sequenced using 454 GS Titanium platform and the resulting ∼1.5 million reads were *de novo* assembled using Newbler).

These libraries, at diluted concentrations, were frozen and thawed frequently during a period of 6 months and yielded consistent qPCR results on expected number of positive wells (data not shown), indicating that the assay is robust and results are reproducible, owing mostly to the use of zero absorbance tubes and exonuclease resistant adapters.

As an alternative to the MGB-Taqman for library quantification, we tested a SYBR green based qPCR assay. Because SYBR green binds to all dsDNA non-specifically, the amplification primer dimers accumulated and gave false signals making them undistinguishable from sample signals (Figure S2). In contrast, the Taqman assay was not affected.

The traditional way of microbial sub-cloning before Sanger sequencing takes days. Our simple and robust assay would enable many laboratories to perform Sanger sequencing using single-molecule polonies, with starting amounts of DNA as low as sub-picogram. It is known that library yield increases with increasing ligation time. Here we used 20 minutes of ligation, for the purpose of rapid screening, and were able to start with 550 femtogram of DNA. With longer ligation time, such as overnight, the starting amount could likely be substantially reduced. In our experimental setting, the background noise signal from adapter dimer became undetectable when the adapter concentration was lowered down to 0.001 µM. Due to the kinetics of a ligation reaction, one needs to balance the concentration of sample and that of adapter, which introduces more noise at higher concentrations but on the other hand renders higher ligation efficiencies.

## Supporting Information

Figure S1 **A continuous demonstration of Table S1.**
(TIF)

Figure S2 **Comparison between Taqman (a) and SYBR green (b) assays.** The primer dimers accumulated and generated signal in the SYBR green assay, and resulting in undistinguishable signal between samples and primer dimers. In contrast, the Taqman assay gave clear signal from the samples.
(TIF)

Data S1 Alignment results (BLASTn ) of thirteen H. pylori reads against NCBI NT database.
(DOC)

Table S1 Expected number of wells having different numbers of DNA molecules (X) using different input DNA template concentrations (λ), in a 96-well plate and a 384-well plate, according Poisson distribution.
(DOC)
