# Supplementary figures and images for: Rapid Screening of Complex DNA Samples by Single-Molecule Amplification and Sequencing
Source: PLoS One. 2011 May 19;6(5):e19723. doi: 10.1371/journal.pone.0019723 (PMC3098247; doi:10.1371/journal.pone.0019723)

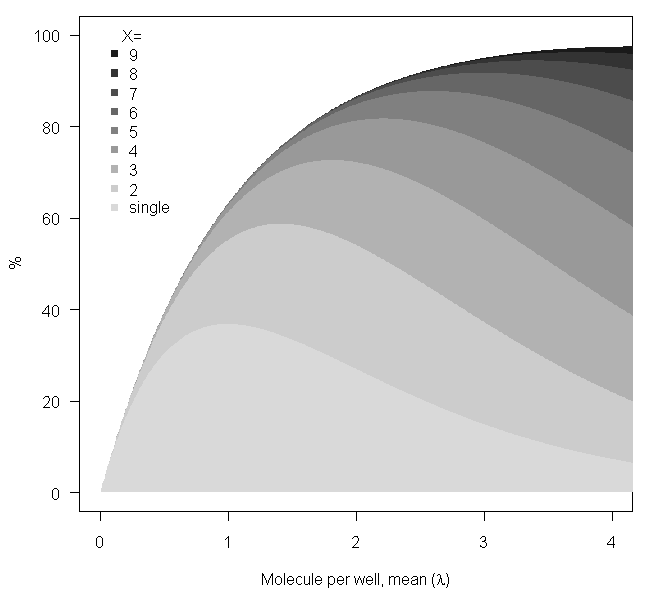

Supplement: Figure S1 — A continuous demonstration of Table S1. (TIF) [file pone.0019723.s001.tif]

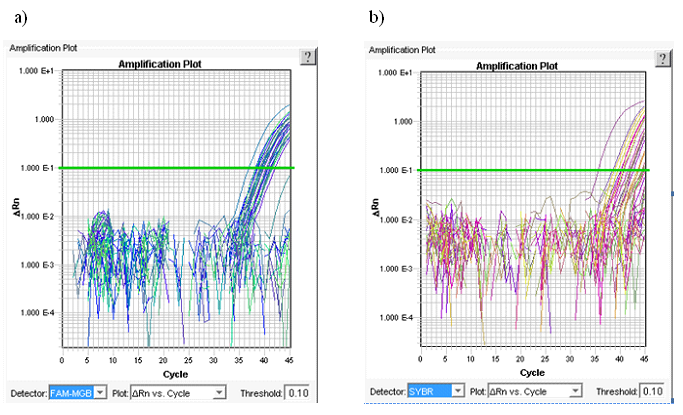

Supplement: Figure S2 — Comparison between Taqman (a) and SYBR green (b) assays. The primer dimers accumulated and generated signal in the SYBR green assay, and resulting in undistinguishable signal between samples and primer dimers. In contrast, the Taqman assay gave clear signal from the samples. (TIF) [file pone.0019723.s002.tif]
