# Supplementary material for: Rapid Screening of Complex DNA Samples by Single-Molecule Amplification and Sequencing
Source: PLoS One. 2011 May 19;6(5):e19723. doi: 10.1371/journal.pone.0019723 (PMC3098247; doi:10.1371/journal.pone.0019723)
Supplement: Data S1 — Alignment results (BLASTn ) of thirteen H. pylori reads against NCBI NT database. (DOC) [file pone.0019723.s003.doc]

# Only the first hit for each query is listed.

Query= A01.ab1

Length=288

ALIGNMENTS

>gb|CP001173.1| Helicobacter pylori G27, complete genome

Length=1652982

Features in this part of subject sequence:

hypothetical protein

Score = 510 bits (265), Expect = 2e-141

Identities = 277/283 (98%), Gaps = 0/283 (0%)

Strand=Plus/Plus

Query 3 AGAAACACGAGCATTTTTGCCCATAAAATCTATCTTATGCGTATCAAAAATAAAGCCTTT 62

|||||||||||||||||||||||||||||||||||||||||||||||||||||||| |||

Sbjct 17758 AGAAACACGAGCATTTTTGCCCATAAAATCTATCTTATGCGTATCAAAAATAAAGCTTTT 17817

Query 63 GAAAATTATGAAGTTCAAGCTGATTCTATTGACGCTTTTTCGTTTTCCTTAAAGCCTTGT 122

||||||||||||||||||||||||||| ||||||||||||||||||| |||||||| |||

Sbjct 17818 GAAAATTATGAAGTTCAAGCTGATTCTGTTGACGCTTTTTCGTTTTCGTTAAAGCCCTGT 17877

Query 123 AAAAGATCGCTTGAAGGCCCCAAAGTCATTGACGCTAGGGAATTGCTTTCAGGGTTTGTA 182

||||||||||||||| ||||||||||||||||||||||||| ||||||||||||||||||

Sbjct 17878 AAAAGATCGCTTGAAAGCCCCAAAGTCATTGACGCTAGGGAGTTGCTTTCAGGGTTTGTA 17937

Query 183 ACAGCCCCACAAATCTTTTGCTCTAACCGCCATAATATTTTATACGTGCGCAGCTTTAAA 242

||||||||||||||||||||||||||||||||||||||||||||||||||||||||||||

Sbjct 17938 ACAGCCCCACAAATCTTTTGCTCTAACCGCCATAATATTTTATACGTGCGCAGCTTTAAA 17997

Query 243 AACGGGTTTGTTTTGAGTCGTTTAAAATGATTTCAAAAccccc 285

|||||||||||||||||||||||||||||||||||||||||||

Sbjct 17998 AACGGGTTTGTTTTGAGTCGTTTAAAATGATTTCAAAACCCCC 18040

Query= B01.ab1

Length=502

ALIGNMENTS

>gb|CP002074.1| Helicobacter pylori PeCan4, complete genome

Length=1629557

Features in this part of subject sequence:

flagellar motor switch protein FliY

hypothetical protein

Score = 742 bits (386), Expect = 0.0

Identities = 445/472 (95%), Gaps = 9/472 (1%)

Strand=Plus/Minus

Query 1 TATTCCTTCCTTCTTTACTTTAATGGTTTAATTTTTCAGTAGCGCTAATCGTTCCTTTTT 60

|||||||||||||||||||||||||||||||||||||||| | |||| ||||| |||||

Sbjct 414736 TATTCCTTCCTTCTTTACTTTAATGGTTTAATTTTTCAGTTGTTCTAAGCGTTCTTTTTT 414677

Query 61 AGTACCAATATCCGTGATCTGAATGCCAAAATTCCCATCCACAATCACCACTTCGCCCTT 120

||| |||||||| |||||||||||||||||||||||||||||||||||||||||||||||

Sbjct 414676 AGTGCCAATATCTGTGATCTGAATGCCAAAATTCCCATCCACAATCACCACTTCGCCCTT 414617

Query 121 AGCGATCACCTTATCATCTACAAGAATTTCCAAAGGGTCATTCACCAATTGATCCAGCTC 180

|||||||||||| |||||||||||||||||||||||||||||||||||||||||||||||

Sbjct 414616 AGCGATCACCTTGTCATCTACAAGAATTTCCAAAGGGTCATTCACCAATTGATCCAGCTC 414557

Query 181 TACCACGCTCCCTATATCCATAGAGACCACATCTTTTAAAATCATCtttttttGCCCGAT 240

||||||||||||||||||||||||||||||||||||||||||||||||||||||||||||

Sbjct 414556 TACCACGCTCCCTATATCCATAGAGACCACATCTTTTAAAATCATCTTTTTTTGCCCGAT 414497

Query 241 GCGCACCTTAACATTCAATTTCACGTCTAAAAGCATGCTGATATTGCGGATTTCTATGTT 300

|||||||||||||||||||||||| |||||||||||||||||||||||||||||||||||

Sbjct 414496 GCGCACCTTAACATTCAATTTCACATCTAAAAGCATGCTGATATTGCGGATTTCTATGTT 414437

Query 301 TTCTAAAGACGCATCATGGGTTTTAACCTCTTCAGTAGCTTCAGTAGCACtctttatcgt 360

||||||||||||||||||||||||||||||||||| |||| |||||| ||||

Sbjct 414436 TTCTAAAGACGCATCATGGGTTTTAACCTCTTCAG---------TAGCGCTCTTTGTCGT 414386

Query 361 ttcttctttttcttctttgtgcgttttttcAAATTGGCGCTCAAAAGACGCCGTAGACAA 420

||||||||||||||||| |||||||||||||||||||||||||||| |||||||| |||

Sbjct 414385 TTCTTCTTTTTCTTCTTGATGCGTTTTTTCAAATTGGCGCTCAAAAGCCGCCGTAGTCAA 414326

Query 421 TAAAATGATTTGGCTTTCTTTGATGGCTTCCATTTTAAAAGAAAACACCATC 472

||| ||||||||||||||||||| |||||||||||||||||||||||||||

Sbjct 414325 TAAGATGATTTGGCTTTCTTTGAGCGCTTCCATTTTAAAAGAAAACACCATC 414274

Query= D01.ab1

Length=411

ALIGNMENTS

>gb|CP001217.1| Helicobacter pylori P12, complete genome

Length=1673813

Features in this part of subject sequence:

fumarate reductase flavoprotein subunit

Score = 750 bits (390), Expect = 0.0

Identities = 404/411 (99%), Gaps = 0/411 (0%)

Strand=Plus/Plus

Query 1 TGTCTTGAATATCCACTTTGTGGTGTAAGGTTTCATTAGCGACCGCATAAAGCATGGTAT 60

|||||||||||||||||||||||||||||| |||||||||||||||||||||||||||||

Sbjct 196601 TGTCTTGAATATCCACTTTGTGGTGTAAGGCTTCATTAGCGACCGCATAAAGCATGGTAT 196660

Query 61 GCCCTGTAGCATCAGCTGTAAAGCATGTGCGCCATTTTTTAGTGCCGCCAAAATCACGGC 120

|||||||||||||||| ||||| |||||||||||||||||||||||||||||||||||||

Sbjct 196661 GCCCTGTAGCATCAGCCGTAAAACATGTGCGCCATTTTTTAGTGCCGCCAAAATCACGGC 196720

Query 121 TTAAAATATAACCATGCCTGTCGTCTCTTTCGGTAATGATAACATGCTCACCATTGACGA 180

||| ||||||||||||||||||||||||||||||||||||||||||||||||||||||||

Sbjct 196721 TTAGAATATAACCATGCCTGTCGTCTCTTTCGGTAATGATAACATGCTCACCATTGACGA 196780

Query 181 CCGCAGGCCTATCGCCCTTTTTAATCCTAGTCCAAGGCACCCCCCAACTGGCCAATTCCC 240

||||||||||||||||||||||||||||||||||||||||||||||||||||||||||||

Sbjct 196781 CCGCAGGCCTATCGCCCTTTTTAATCCTAGTCCAAGGCACCCCCCAACTGGCCAATTCCC 196840

Query 241 TAATGGCTTTAGGAGCGGTGGTTACAAACATTCTAGCCACTTGCTGATCGCACCCCCAAT 300

|||||||||||||||| |||||||||||||||||||||||||||||||||||||||||||

Sbjct 196841 TAATGGCTTTAGGAGCAGTGGTTACAAACATTCTAGCCACTTGCTGATCGCACCCCCAAT 196900

Query 301 CGCTCCCCTTAACCGTGTCTAAAAAGTGCAGATCTTCATTATCGCCCTCGCtttttttAG 360

|||||||||||||||||||||||||||||| |||||||||||||||||||||||||||||

Sbjct 196901 CGCTCCCCTTAACCGTGTCTAAAAAGTGCAAATCTTCATTATCGCCCTCGCTTTTTTTAG 196960

Query 361 CGTTCGCAAGACTCGCTTGCATGCCCCCTTGAGCGGCTGCAGAGTGCGAAC 411

|||||||||| ||||||||||||||||||||||||||||||||||||||||

Sbjct 196961 CGTTCGCAAGGCTCGCTTGCATGCCCCCTTGAGCGGCTGCAGAGTGCGAAC 197011

Query= F01.ab1

Length=151

ALIGNMENTS

>gb|CP002074.1| Helicobacter pylori PeCan4, complete genome

Length=1629557

Features in this part of subject sequence:

3-methyladenine DNA glycosylase

Score = 250 bits (130), Expect = 3e-63

Identities = 142/148 (96%), Gaps = 0/148 (0%)

Strand=Plus/Plus

Query 4 GGTGGATAAATACAGCTATCtttttttaaaaaaaaTAGGCATAGAGATAGAAGATTATGA 63

|||||||||||||||||||||||||||||||||| |||||||||||||||||||||||||

Sbjct 596267 GGTGGATAAATACAGCTATCTTTTTTTAAAAAAATTAGGCATAGAGATAGAAGATTATGA 596326

Query 64 CGAATTGCCACAtttttttGAAAAAGGCGTTCCAGAGAATTTAAATGCCCCTTTAGCGCT 123

|||||||| ||||||||||||||||||||||| ||||||||||||| || ||||||||||

Sbjct 596327 CGAATTGCAACATTTTTTTGAAAAAGGCGTTCAAGAGAATTTAAATTCCGCTTTAGCGCT 596386

Query 124 TTATAAAAACACCATTTCTTTAGCGCAA 151

|||| |||||||||||||||||||||||

Sbjct 596387 TTATGAAAACACCATTTCTTTAGCGCAA 596414

Query= G01.ab1

Length=525

ALIGNMENTS

>gb|AE000511.1| Helicobacter pylori 26695, complete genome

Length=1667867

Features in this part of subject sequence:

acriflavine resistance protein (acrB)

Score = 908 bits (472), Expect = 0.0

Identities = 502/517 (98%), Gaps = 0/517 (0%)

Strand=Plus/Plus

Query 1 AGATTGCTGGAGCGAATGAAATTGAAATCGTGGATAGGGTGTATGAAGCTTTAAAACACA 60

||||||| ||||||||||||||||||||||| ||||||||||||||||||||||| | ||

Sbjct 644295 AGATTGCCGGAGCGAATGAAATTGAAATCGTAGATAGGGTGTATGAAGCTTTAAAGCGCA 644354

Query 61 TTCAAGCCATTAGCCCTAGCTATGAAATCAGACCCTTTTTAGACACCACGGGCTATATCC 120

|||||||||||||||||| |||||||||||||||||||||||||||||||||||||||||

Sbjct 644355 TTCAAGCCATTAGCCCTAACTATGAAATCAGACCCTTTTTAGACACCACGGGCTATATCC 644414

Query 121 GCACCTCTATTGAAGACGTGAAATTTGATCTAGTCTTAGGGGCGATTTTAGCGGTTTTAG 180

|||||||||||||||||||||||||||||||||| |||||||||||||||||||||||||

Sbjct 644415 GCACCTCTATTGAAGACGTGAAATTTGATCTAGTTTTAGGGGCGATTTTAGCGGTTTTAG 644474

Query 181 TGGTGTTCGCGTTCTTGCGTAACGGCACGATCACCCTTGTTTCAGCGATCTCTATCCCTA 240

||||||| ||||||||||||||||||||||||||||| ||||||||||||||||||||||

Sbjct 644475 TGGTGTTTGCGTTCTTGCGTAACGGCACGATCACCCTCGTTTCAGCGATCTCTATCCCTA 644534

Query 241 TTTCTATCATGGGGACTTTTGCGCTCATTCAATGGATGGGCTTTTCATTAAACATGCTCA 300

|||||||||||||||||||||||||||| |||||||||||||||||||||||||||||||

Sbjct 644535 TTTCTATCATGGGGACTTTTGCGCTCATCCAATGGATGGGCTTTTCATTAAACATGCTCA 644594

Query 301 CCATGGTGGCTTTAACGCTGGCGATAGGGATTATCATTGATGATGCGATCGTGGTGATTG 360

||||||||||||||||| ||||||||||||||||||||||||||||||||||||||||||

Sbjct 644595 CCATGGTGGCTTTAACGTTGGCGATAGGGATTATCATTGATGATGCGATCGTGGTGATTG 644654

Query 361 AAAACATCCATAAAAAGCTAGAAATGGGTATGAGTAAACAAAAAGCGAGCTATGAGGGGG 420

|||||||||||||||||||||||||||| |||||||||| ||||||||||||||||||||

Sbjct 644655 AAAACATCCATAAAAAGCTAGAAATGGGCATGAGTAAACGAAAAGCGAGCTATGAGGGGG 644714

Query 421 TGAGAGAAATTGGCTTTGCTTTAGTGGCGATTTCAGCAATGCTGCTCTCTGTTTTTGTGC 480

|||||||||||||||||||| |||||||||||||||| ||||||||||||||||||||||

Sbjct 644715 TGAGAGAAATTGGCTTTGCTCTAGTGGCGATTTCAGCGATGCTGCTCTCTGTTTTTGTGC 644774

Query 481 CTATAGGGAACATGAAAGGCATTATTGGGCGCTTTTT 517

||||||||||||||||||||||||||||||| |||||

Sbjct 644775 CTATAGGGAACATGAAAGGCATTATTGGGCGTTTTTT 644811

Query= H01.ab1

Length=298

ALIGNMENTS

>emb|FN598874.1| Helicobacter pylori B8 complete genome

Length=1673997

Features in this part of subject sequence:

ATP-dependent Lon protease

Score = 479 bits (249), Expect = 4e-132

Identities = 257/261 (99%), Gaps = 0/261 (0%)

Strand=Plus/Minus

Query 1 GCTTGAATGTTTTCTTTATCGTATTCTAAATATTCAATAGGGCTTATTTGAGCTTCTAAA 60

||||||||||||||||||||||||||||||||||||||||||||||||||||||||||||

Sbjct 1517961 GCTTGAATGTTTTCTTTATCGTATTCTAAATATTCAATAGGGCTTATTTGAGCTTCTAAA 1517902

Query 61 AAGCCTTGCTCGTTTTCTTTAGCAGGCTCTAAAATACGCCCCTTAGCGATGCCATTAAAG 120

||||||||||||||||||||||||||||||||||||||||||||||||||||||||||||

Sbjct 1517901 AAGCCTTGCTCGTTTTCTTTAGCAGGCTCTAAAATACGCCCCTTAGCGATGCCATTAAAG 1517842

Query 121 AGCAATTTCACGCGCCCATTAGGCATGTTGGCTTCACGCATAATAGATCCAATCACCCCC 180

||||||||||||||||||||||||||||||||||||||||||||||||||||||||||||

Sbjct 1517841 AGCAATTTCACGCGCCCATTAGGCATGTTGGCTTCACGCATAATAGATCCAATCACCCCC 1517782

Query 181 ACATCATAATAAGGGGCTTCATTGTCATTCAATTTGTCTTTTTGGCACGCAATAAAGACT 240

||||||||||||||||||||||| || |||||||||||||||||||| ||||||||||||

Sbjct 1517781 ACATCATAATAAGGGGCTTCATTATCGTTCAATTTGTCTTTTTGGCATGCAATAAAGACT 1517722

Query 241 AATGATTTATTGTTTTTAGCG 261

|||||||| ||||||||||||

Sbjct 1517721 AATGATTTGTTGTTTTTAGCG 1517701

Query= A02.ab1

Length=412

ALIGNMENTS

>gb|CP002334.1| Helicobacter pylori Lithuania75, complete genome

Length=1624644

Features in this part of subject sequence:

DNA repair protein RadA

Score = 673 bits (350), Expect = 0.0

Identities = 368/377 (98%), Gaps = 0/377 (0%)

Strand=Plus/Minus

Query 1 TAATCACAGGCCAATTGATTTCATTGAGCAGATACAATTCTTTTTCTACGCAATCCAATC 60

|||||||||||||||||||||||||||||||||||||||||||||||| |||||||||||

Sbjct 222983 TAATCACAGGCCAATTGATTTCATTGAGCAGATACAATTCTTTTTCTATGCAATCCAATC 222924

Query 61 TAGTGGCGCGCATTTTAATCTGGCTCAAGCTCTCTTCCCCGCTCACATACAAAACCTTTT 120

|| |||||||||||||||||||||||||||||||||||||||||||||||||||||||||

Sbjct 222923 TAATGGCGCGCATTTTAATCTGGCTCAAGCTCTCTTCCCCGCTCACATACAAAACCTTTT 222864

Query 121 GCTGGTTTTTGGCTAAGCCAGAAGCCACTTTTAAAAGCAGAGTGGATTTCCCCACCCCAG 180

||||||||||||||||||||||||||||||||||||||||||||||||||||||||||||

Sbjct 222863 GCTGGTTTTTGGCTAAGCCAGAAGCCACTTTTAAAAGCAGAGTGGATTTCCCCACCCCAG 222804

Query 181 GACTcccccccACTAAATACAACCCTCCTTTAGCGATCCCCCCACCAAAAACAATATCCA 240

||||||||||||||||||||||||| |||||||||||||||||||| |||||||||||||

Sbjct 222803 GACTCCCCCCCACTAAATACAACCCCCCTTTAGCGATCCCCCCACCTAAAACAATATCCA 222744

Query 241 ATTCGCTTTGAGTGGAAGAAAACTTAATCACTCCTTCTTGCTCAATTGCAGCGATAGAAA 300

|||||||||||||||||||||||||||||||| |||| ||||||||||||||||||||||

Sbjct 222743 ATTCGCTTTGAGTGGAAGAAAACTTAATCACTTCTTCATGCTCAATTGCAGCGATAGAAA 222684

Query 301 CGCTTTTTTGCACTTGTGGGATCGGTTTTTTAAGCGCGTGTAAAACTTCCTTTTGAGCTT 360

||||||||||| ||| ||||| ||||||||||||||||||||||||||||||||||||||

Sbjct 222683 CGCTTTTTTGCGCTTTTGGGAGCGGTTTTTTAAGCGCGTGTAAAACTTCCTTTTGAGCTT 222624

Query 361 GGTTCAATTCTATAAAA 377

|||||||||||||||||

Sbjct 222623 GGTTCAATTCTATAAAA 222607

Query= B02.ab1

Length=421

ALIGNMENTS

>gb|CP002076.1| Helicobacter pylori Cuz20, complete genome

Length=1635449

Features in this part of subject sequence:

PARA protein

Score = 763 bits (397), Expect = 0.0

Identities = 411/418 (99%), Gaps = 0/418 (0%)

Strand=Plus/Minus

Query 4 GATAGCCAAAAGAGTTTGGAAGTGTTTAATAATATTAGAAGTGAAACAAGTTTGCCCAAT 63

||||| |||||||||||||||||||| |||||||||||||||||||||||||||||||||

Sbjct 249523 GATAGTCAAAAGAGTTTGGAAGTGTTCAATAATATTAGAAGTGAAACAAGTTTGCCCAAT 249464

Query 64 TTCACGCTCTTTAACCGCACAGGCAATATCACAGACACCTTAAAACAGATGATGGATAAA 123

|||||||||||||| |||||||||||||||||||||||||||||||||||||||||||||

Sbjct 249463 TTCACGCTCTTTAATCGCACAGGCAATATCACAGACACCTTAAAACAGATGATGGATAAA 249404

Query 124 TACGAATACATCCTTATTGATACTAAGGGCGAACATTCCAAAGAAAGCCAAAGGGCTATG 183

|||||||||||||||||||||||||||||||||||||| |||||||||||||||||||||

Sbjct 249403 TACGAATACATCCTTATTGATACTAAGGGCGAACATTCTAAAGAAAGCCAAAGGGCTATG 249344

Query 184 TTATTGAGCGATTGGGTGCTAATACCCACCACGCCAAGCCAACTAGACACAGCGGTGCTA 243

||||||||||||||||||||||||||||||||||||||||||||||||||||||||||||

Sbjct 249343 TTATTGAGCGATTGGGTGCTAATACCCACCACGCCAAGCCAACTAGACACAGCGGTGCTA 249284

Query 244 TTAGACATGCTAGAAAGGATTAGAGACATCCAAGCGTTGAATGAAAACTTAAAAGCTTGT 303

||||||||||||||||||||||| ||||||||||||||||||||||||||||||||||||

Sbjct 249283 TTAGACATGCTAGAAAGGATTAGGGACATCCAAGCGTTGAATGAAAACTTAAAAGCTTGT 249224

Query 304 ATTGTGATGAACCGCATCCCTACTATCCCCACTCTTAAAGAGAAAAAAGCTCTCGTTGAT 363

|||||||||||||||||||||||||||||||||||||||||||||||||||||| |||||

Sbjct 249223 ATTGTGATGAACCGCATCCCTACTATCCCCACTCTTAAAGAGAAAAAAGCTCTCATTGAT 249164

Query 364 TTTATCAACCAAAATAACGCTAATGAAAGCGTGTTTTTAATGGATAATATATTAAGCG 421

|||||||||||||||||||||||||||||||||||||||||||||||| |||||||||

Sbjct 249163 TTTATCAACCAAAATAACGCTAATGAAAGCGTGTTTTTAATGGATAATGTATTAAGCG 249106

Query= C02.ab1

Length=378

ALIGNMENTS

>gb|CP000241.1| Helicobacter pylori HPAG1, complete genome

Length=1596366

Features in this part of subject sequence:

putative zinc protease

Score = 569 bits (296), Expect = 3e-159

Identities = 307/310 (99%), Gaps = 1/310 (0%)

Strand=Plus/Minus

Query 1 CCATTGGTTTTATGGACGATATTCAAAAA-GGACTTTAAAAGACATTAAAAAATTCCATT 59

|||||||||||||||||||||||||||| ||||||||||||||||||||||||||||||

Sbjct 443171 CCATTGGTTTTATGGACGATATTCAAAACTGGACTTTAAAAGACATTAAAAAATTCCATT 443112

Query 60 CGCTCTATTATCAGCCTAAAAACGCTATTATTTTAGTGGTGGGCGATGTCAATTCCCAAA 119

||||||||||||||||||||||||||||||||||||||||||||||||||||||||||||

Sbjct 443111 CGCTCTATTATCAGCCTAAAAACGCTATTATTTTAGTGGTGGGCGATGTCAATTCCCAAA 443052

Query 120 AGGTTTTTGAATTGAGTAAAAAGCATTTTGAATCCTTAAAAAACCTTGATGAAAAAGCTA 179

||||||||||||||||||||||||||||||||||||||||||||||||||||||||||||

Sbjct 443051 AGGTTTTTGAATTGAGTAAAAAGCATTTTGAATCCTTAAAAAACCTTGATGAAAAAGCTA 442992

Query 180 TCCCCACCCCTTACATGAAAGAGCCTAAACAAGATGGAGCCAGAACGGCAGTCGTGCATA 239

|||||||||||||||||||||||||||| |||||||||||||||||||||||||||||||

Sbjct 442991 TCCCCACCCCTTACATGAAAGAGCCTAAGCAAGATGGAGCCAGAACGGCAGTCGTGCATA 442932

Query 240 AAGATGGGGTCCATTTAGAATGGGTAGCGTTAGGGTATAAAGTGCCTGCTTTCAAGCATA 299

||||||||||||||||||||||||||||||||||||||||||||||||||||||||||||

Sbjct 442931 AAGATGGGGTCCATTTAGAATGGGTAGCGTTAGGGTATAAAGTGCCTGCTTTCAAGCATA 442872

Query 300 AAGATCAAGT 309

||||||||||

Sbjct 442871 AAGATCAAGT 442862

Query= E02.ab1

Length=118

ALIGNMENTS

>gb|CP001680.1| Helicobacter pylori 52, complete genome

Length=1568826

Features in this part of subject sequence:

ribonucleotide-diphosphate reductase subunit alpha

Score = 219 bits (114), Expect = 6e-54

Identities = 116/117 (99%), Gaps = 0/117 (0%)

Strand=Plus/Plus

Query 1 ACCATGCGATTGACACGAGCGCGAATCTAGCGAAAGAAAAAGGGGTTTATAAGGATTTTG 60

|||||||||||||||||||||||||| |||||||||||||||||||||||||||||||||

Sbjct 691970 ACCATGCGATTGACACGAGCGCGAATTTAGCGAAAGAAAAAGGGGTTTATAAGGATTTTG 692029

Query 61 AAAATTCAGAATGGAGTAAGGGGATTTTCCCTATTGACAAAGCCAATAATGAAGCCT 117

|||||||||||||||||||||||||||||||||||||||||||||||||||||||||

Sbjct 692030 AAAATTCAGAATGGAGTAAGGGGATTTTCCCTATTGACAAAGCCAATAATGAAGCCT 692086

Query= F02.ab1

Length=453

ALIGNMENTS

>gb|CP000241.1| Helicobacter pylori HPAG1, complete genome

Length=1596366

Features in this part of subject sequence:

ribonucleoside-diphosphate reductase 1 alpha subunit

Score = 777 bits (404), Expect = 0.0

Identities = 412/416 (99%), Gaps = 0/416 (0%)

Strand=Plus/Plus

Query 1 ATAAATGGGTTCAATCGTTTGGGTTGTGCCTACTAAAATAGAAATGGAGCTTGTGGGAGC 60

||||||||||||||||||||||||||||||||||||||||||||||||||||||||||||

Sbjct 692690 ATAAATGGGTTCAATCGTTTGGGTTGTGCCTACTAAAATAGAAATGGAGCTTGTGGGAGC 692749

Query 61 GATCGCCATTAAATAGCCATTACGCATGCCATTAGCCTTGACTTTTTCTCTCAAACCTTG 120

||||||||||||||||||||||||||||||||||||||||||||||||||||||||||||

Sbjct 692750 GATCGCCATTAAATAGCCATTACGCATGCCATTAGCCTTGACTTTTTCTCTCAAACCTTG 692809

Query 121 CCAATCGCAAGCGTGATTGAAAAGCCCTTTTTCGGTGAGCTTTAAGGCTTCATTATTGGC 180

||||||||||||||||||||||||||||||||||||||||||||||||||||||||||||

Sbjct 692810 CCAATCGCAAGCGTGATTGAAAAGCCCTTTTTCGGTGAGCTTTAAGGCTTCATTATTGGC 692869

Query 181 TTTGTCAATAGGGAAAATCCCCTTACTCCATTCTGAATTTTCAAAATCCTTATAAACCCC 240

||| ||||| ||||||||||||||||||||||||||||||||||||||||||||||||||

Sbjct 692870 TTTATCAATGGGGAAAATCCCCTTACTCCATTCTGAATTTTCAAAATCCTTATAAACCCC 692929

Query 241 TTTTTCTTTCGCTAGATTCGCGCTCGTGTCAATCGCATGGTAGCTGATTTGCTCCATTAA 300

||||||||||||||||||||||||||||||||||||||||||||||||||||||||||||

Sbjct 692930 TTTTTCTTTCGCTAGATTCGCGCTCGTGTCAATCGCATGGTAGCTGATTTGCTCCATTAA 692989

Query 301 AGCGTCAATTTTTTCTAAATGCTCTTTAGACCCCCAAGCGATTTGGTGTTCTGCGAGCAT 360

||||||||||||||||||||||||||||||||||||||||||||| ||||||||||||||

Sbjct 692990 AGCGTCAATTTTTTCTAAATGCTCTTTAGACCCCCAAGCGATTTGATGTTCTGCGAGCAT 693049

Query 361 TTGCGCTTCACCCATAACCCCTAACCCTATGGCCCTATTTTGTAAATTAGTGGCTT 416

|||||||||||||||||||||||||||||||||||||||||||||||| |||||||

Sbjct 693050 TTGCGCTTCACCCATAACCCCTAACCCTATGGCCCTATTTTGTAAATTGGTGGCTT 693105

Query= G02.ab1

Length=488

ALIGNMENTS

>emb|FN598874.1| Helicobacter pylori B8 complete genome

Length=1673997

Features in this part of subject sequence:

two-component system, chemotaxis family, sensor kinase CheA

Score = 833 bits (433), Expect = 0.0

Identities = 475/491 (97%), Gaps = 3/491 (0%)

Strand=Plus/Plus

Query 1 CTTTTTCTTTA-GCATCACAGGGTCTAATCCCTTGT--TTAGCTTGGATTTTAATCACAA 57

||||||||||| |||||||||||||||| |||||| | | | ||||||||||||||

Sbjct 409203 CTTTTTCTTTAAGCATCACAGGGTCTAACCCCTTGCCATCATCAGAGATTTTAATCACAA 409262

Query 58 TGTGGTTACCCTCATTATACGCCCTCAATTGCACCTTACCGGTTTCAGGCTTGTTAAGCC 117

|||||||||||||||||||||| ||||||||||| |||||||||||||||||||||||||

Sbjct 409263 TGTGGTTACCCTCATTATACGCGCTCAATTGCACTTTACCGGTTTCAGGCTTGTTAAGCC 409322

Query 118 TTCTTCTTTCTTCTAAAGGCTCAATCCCATGATCGCATGAGTTGCGGATAATGTGGATGA 177

||||||||||||||||||||||||||||||||||||||||||||||||||||||||||||

Sbjct 409323 TTCTTCTTTCTTCTAAAGGCTCAATCCCATGATCGCATGAGTTGCGGATAATGTGGATGA 409382

Query 178 GCGGATCGCCAATCTCTTCTACAATGGATTTGTCCAATTCGGTTTCTTCGCCCTCAATGA 237

|||||||||||||||||||||||||||||||||| |||||||||||||||||||||||||

Sbjct 409383 GCGGATCGCCAATCTCTTCTACAATGGATTTGTCTAATTCGGTTTCTTCGCCCTCAATGA 409442

Query 238 TTAATTCAATGCTCTTGCCTAATTCCCGGCTCAAATCCCTTACCATGCGAGGGAATTTAT 297

||||||||||||||||||||||||||||||||||||||||||||||||||||||||||||

Sbjct 409443 TTAATTCAATGCTCTTGCCTAATTCCCGGCTCAAATCCCTTACCATGCGAGGGAATTTAT 409502

Query 298 TGAACACTTTGCCCACTGGTTGCATCCTGGTTTTCATCACCGCAAGTTGCAAGTCTGTCG 357

||||||||||||||||||||||||||| ||||||||||||||||||||||||||||||||

Sbjct 409503 TGAACACTTTGCCCACTGGTTGCATCCGGGTTTTCATCACCGCAAGTTGCAAGTCTGTCG 409562

Query 358 TTACCGCTGAAATAGAAGAAACCACCTGGTTTAATTCCTCTAAAAACTTTTCCCCATCAT 417

||||||||||||||||||||||||||||||||||||||||||||||||||||||||||||

Sbjct 409563 TTACCGCTGAAATAGAAGAAACCACCTGGTTTAATTCCTCTAAAAACTTTTCCCCATCAT 409622

Query 418 AGCGTTCTTCCACATCGCTATAAATCCTGATCAAGCGATTCTTTCCTAACACAAGCTCAC 477

|||||||||||||||||| ||||||||||||||||||||| |||||||||||||||||||

Sbjct 409623 AGCGTTCTTCCACATCGCCATAAATCCTGATCAAGCGATTTTTTCCTAACACAAGCTCAC 409682

Query 478 CGATTAAATTC 488

|||||||||||

Sbjct 409683 CGATTAAATTC 409693

Query= H02.ab1

Length=248

ALIGNMENTS

>gb|AE001439.1| Helicobacter pylori J99, complete genome

Length=1643831

Features in this part of subject sequence:

putative Outer membrane protein

Score = 408 bits (212), Expect = 1e-110

Identities = 232/242 (96%), Gaps = 0/242 (0%)

Strand=Plus/Minus

Query 1 CCATTCAATTGCCCCCCATAGTTAGGCAAGAAATTCAAACCTTTATTGTTTAAATCCAAA 60

|||||||||||||||||||| |||||||||||||||||||||||||||||||||||||||

Sbjct 1201279 CCATTCAATTGCCCCCCATAATTAGGCAAGAAATTCAAACCTTTATTGTTTAAATCCAAA 1201220

Query 61 TAGGGGGCGACCCAATTGGTGATAAAATCCCCATACGATACGGGGTTACTAAGCAAGCTT 120

||||||||||||||||||||||| ||||||||||||| ||||| ||||||||||||||||

Sbjct 1201219 TAGGGGGCGACCCAATTGGTGATGAAATCCCCATACGCTACGGTGTTACTAAGCAAGCTT 1201160

Query 121 TCCCCATAGTTGATCGCGTCTTGCATCTGGTTATAGCTTGAAGTACCTGTCAGTTTAGCG 180

||||||||||||||||| |||||||||||||||||||||||||| ||| |||||||||||

Sbjct 1201159 TCCCCATAGTTGATCGCATCTTGCATCTGGTTATAGCTTGAAGTGCCTATCAGTTTAGCG 1201100

Query 181 GCTTTATTAGCAAAATTTTGCATGCCTGAAAAAATCATGGAAGCGGTGATGCCATTAGCG 240

|||||||| || |||||||||||||||||||||||||| |||||||||||||||||||||

Sbjct 1201099 GCTTTATTGGCGAAATTTTGCATGCCTGAAAAAATCATAGAAGCGGTGATGCCATTAGCG 1201040

Query 241 AT 242

||

Sbjct 1201039 AT 1201038

Database: All GenBank+EMBL+DDBJ+PDB sequences (but no EST, STS,

GSS,environmental samples or phase 0, 1 or 2 HTGS sequences)

Posted date: Feb 6, 2011 4:39 PM

Number of letters in database: 748,815,153

Number of sequences in database: 14,763,360

Lambda K H

1.33 0.621 1.12

Gapped

Lambda K H

1.33 0.621 1.12

Matrix: blastn matrix:1 -2

Gap Penalties: Existence: 5, Extension: 2

Number of Sequences: 14763360

Number of Hits to DB: 95527790

Number of extensions: 448235

Number of successful extensions: 448235

Number of sequences better than 10: 23

Number of HSP's better than 10 without gapping: 0

Number of HSP's gapped: 448233

Number of HSP's successfully gapped: 40

Length of database: 35108553521

A: 0

X1: 11 (21.1 bits)

X2: 15 (28.8 bits)

X3: 52 (100.0 bits)

S1: 9 (18.0 bits)
