# Supplementary material for: Rapid Screening of Complex DNA Samples by Single-Molecule Amplification and Sequencing
Source: PLoS One. 2011 May 19;6(5):e19723. doi: 10.1371/journal.pone.0019723 (PMC3098247; doi:10.1371/journal.pone.0019723)
Supplement: Table S1 — Expected number of wells having different numbers of DNA molecules (X) using different input DNA template concentrations (λ), in a 96-well plate and a 384-well plate, according Poisson distribution. (DOC) [file pone.0019723.s004.doc]

|  | **Input DNA template concentration, mean copy / well (λ)** | | | | | | |
| --- | --- | --- | --- | --- | --- | --- | --- |
| **X** | **0.1** | **0.16** | **0.3** | **0.5** | **1** | **2** | **4** |
| For a 96-well plate | | | | | |  |  |
| **One** | 8.7 | 13.1 | 21.3 | 29.1 | 35.3 | 26.0 | 7.0 |
| **Two** | 0.4 | 1.0 | 3.2 | 7.3 | 17.7 | 26.0 | 14.1 |
| **Three** | 0.0 | 0.1 | 0.3 | 1.2 | 5.9 | 17.3 | 18.8 |
| **Four** | 0.0 | 0.0 | 0.0 | 0.2 | 1.5 | 8.7 | 18.8 |
| **Five+** | 0.0 | 0.0 | 0.0 | 0.0 | 0.4 | 5.1 | 35.6 |
|  |  |  |  |  |  |  |  |
| For a 384-well plate | | | | | | | |
| **One** | 34.7 | 52.4 | 85.3 | 116.5 | 141.3 | 103.9 | 28.1 |
| **Two** | 1.7 | 4.2 | 12.8 | 29.1 | 70.6 | 103.9 | 56.3 |
| **Three** | 0.1 | 0.2 | 1.3 | 4.9 | 23.5 | 69.3 | 75.0 |
| **Four** | 0.0 | 0.0 | 0.1 | 0.6 | 5.9 | 34.6 | 75.0 |
| **Five+** | 0.0 | 0.0 | 0.0 | 0.1 | 1.4 | 20.2 | 142.5 |
|  |  |  |  |  |  |  |  |
| % single-molecule (one) among positive wells | | | | | |  |  |
|  | 96% | 92% | 86% | 77% | 58% | 31% | 7% |
